# Exploring the Intricate Links between Adenotonsillar Hypertrophy, Mouth Breathing, and Craniofacial Development in Children with Sleep-Disordered Breathing: Unraveling the Vicious Cycle

**DOI:** 10.3390/children10081426

**Published:** 2023-08-21

**Authors:** Luana Nosetti, Marco Zaffanello, Francesca De Bernardi di Valserra, Daniela Simoncini, Giulio Beretta, Pietro Guacci, Giorgio Piacentini, Massimo Agosti

**Affiliations:** 1 Pediatric Sleep Disorders Center, Division of Pediatrics, “F. Del Ponte” Hospital, University of Insubria, 21100 Varese, Italy; luana.nosetti@uninsubria.it (L.N.); daniela.simoncini@libero.it (D.S.); gberetta8@studenti.uninsubria.it (G.B.); pguacci@studenti.uninsubria.it (P.G.); giorgio.piacentini@univr.it (G.P.); 2 Department of Surgery, Dentistry, Pediatrics and Gynecology, University of Verona, 37100 Verona, Italy; 3 Division of Otorhinolaryngology, Department of Biotechnologies and Life Sciences, University of Insubria, Ospedale di Circolo e Fondazione Macchi, 21100 Varese, Italy; francesca.debernardi@ospedale.varese.it; 4 Department of Medicine and Surgery, University of Insubria, 21100 Varese, Italy; massimo.agosti@uninsubria.it

**Keywords:** adenotonsillar hypertrophy, adenotonsillectomy, children, craniofacial alteration, craniofacial development, craniofacial anomaly, obstructive sleep apnea, sleep-disordered breathing

## Abstract

Adenotonsillar hypertrophy has been well-acknowledged as the primary instigator of sleep-disordered breathing in the pediatric population. This condition spans a spectrum, from typical age-related growth that the immune system influences to persistent pathological hypertrophy. Reduction in air spaces, metabolic changes, neurobehavioral alterations, and chronic inflammation characterizes the latter form. As the go-to treatment, adenotonsillectomy has proven effective. However, it is not a guarantee for all patients, leaving us without reliable predictors of treatment success. Evidence suggests a connection between adenotonsillar hypertrophy and specific oral breathing patterns resulting from craniofacial development. This finding implies an intricate interdependence between the two, hinting at a self-sustaining vicious cycle that persists without proper intervention. The theories regarding the relationship between craniofacial conformation and sleep-disordered breathing have given rise to intriguing perspectives. In particular, the “gracilization theory” and the “gravitational hypothesis” have provided fascinating insights into the complex interaction between craniofacial conformation and SDB. Further investigation is crucial to unraveling the underlying pathophysiological mechanisms behind this relationship. It is also vital to explore the risk factors linked to adenotonsillectomy failure, study the long-term effects of adenotonsillar hypertrophy on craniofacial growth, and devise innovative diagnostic techniques to detect upper airway compromise early. Moreover, to assess their efficacy, we must delve into novel therapeutic approaches for cases that do not respond to traditional treatment, including positional therapy and orofacial myofunctional therapy. Though complex and unpredictable, these challenges promise to enhance our understanding and treatment of adenotonsillar hypertrophy and its related complications in children. By taking on this task, we can pave the way for more effective and targeted interventions, ultimately improving affected individuals’ well-being and quality of life.

## 1. Introduction

Obstructive sleep apnea (OSA) is a respiratory disorder that occurs during sleep, characterized by repetitive episodes of partial or complete obstruction in the upper airway, leading to impaired breathing [1]. This condition is quite common among children, with reported prevalence rates of 27% for snoring and 5.7% for OSA, according to various studies [2,3,4]. However, it is worth noting that the prevalence and severity of sleep-disordered breathing (SDB) can vary significantly among different populations studied.

The clinical manifestations of OSA have profound implications, affecting various aspects of a child’s health, including behavior, neurocognitive functions, cardiovascular health, and overall quality of life [5,6]. Polysomnography (PSG) is widely acknowledged as the definitive method for assessing SDB [7].

Adenotonsillar hypertrophy (ATH) has a strong association with SDB in children [8,9]. ATH is strongly associated with SDB in children [10]. However, ATH not only plays a critical role in OSA but also contributes to the enlargement of the soft palate. Specific skeletal patterns can influence the development and progression of upper airway obstruction [11].

Facial skeletal abnormalities can occur in varying percentages of pediatric children, even those considered otherwise healthy. Children with SDB related to mouth breathing are likelier to exhibit a convex facial profile, increased lower facial height, mandibular retrusion, tonsillar hypertrophy, and mouth breathing [12]. SDB related to mouth breathing can affect craniofacial growth from a dental perspective [2,3,4]. The prevalence of Angle malocclusion varies significantly, with estimates ranging from 6.5% to 51.9% [13]. However, the exact prevalence of malocclusion may vary depending on the diagnostic methods used.

Dental treatment, possibly in combination with evaluation by an otolaryngologist and weight control, can help alleviate respiratory issues and their clinical consequences [2,3,4].

The aims of this comprehensive review are outlined as follows: (1) to explore the implications of ATH on facial morphology and appraise its influence on facial shape, (2) to investigate the correlation between craniofacial conformation and the severity of SDB in individuals with ATH, and (3) to explore the two theories that attempt to explain the complex relationship between craniofacial conformation and SDB in the context of ATH.

## 2. Adenoids and Tonsils: Unraveling the Complexities of Airway Health

From birth, the adenoids are an integral part of our respiratory system. They gradually increase in size as we age, reaching their peak dimensions at around 6 to 7 years old. Subsequently, these critical tissues undergo a process of atrophy, ultimately merging with the mucosa of the nasopharyngeal wall [14]. Traditionally, the behavior of the pharynx has been attributed to Bernoulli’s theorem. However, it is essential to recognize that our airway is not a singular tube; instead, it takes the form of a bifurcated structure [15]. When an entrance to the airway, such as the nasal valve, becomes obstructed, the air is re-directed through the oral route. Despite this, the negative pressure continues to impact the nasopharynx [16]. This pressure correlates with nasal resistance and can trigger inflammation in the mucous membranes of the nasopharynx, initiating an intricate inflammatory cascade [17].

The Enigma of Adenoids and Tonsils:

Positioned at the crossroads of the respiratory and digestive systems, the adenoids and tonsils serve as continuous sentinels, exposed to an array of microbial antigens, both viral and bacterial, along with allergens. Due to their strategic location, these tissues are often regarded as reservoirs of various viruses and bacteria, actively participating in immune activity. Due to chronic or recurrent infections, hypertrophy of the adenoids and tonsils can occur, increasing lymphoid elements. The condition referred to as ATH can result in a variety of complications, including middle ear infections, rhinosinusitis, obstructive sleep apnea syndrome (OSAS), and swallowing difficulties [18,19]. ATH can also manifest as a physiological response and is potentially reversible once the underlying pathological agent is eliminated [20].

The Complexities of Airway Constriction:

As we delve into the intricacies of airway constriction, we encounter the retro-palatal area, which constitutes the overlapping space between the tonsils and adenoids. Notably, this area exhibits the smallest cross-sectional dimensions among all pharyngeal airways [21]. The initial narrowing takes place in the adenoid-palatal space, followed by the hypertrophy of the anterior region, ultimately leading to the closure of the adenoid-canal area [22]. Furthermore, the interplay of abnormal neuromotor tone can contribute to airway narrowing and subsequent collapse during sleep [23].

Challenges in Treatment:

Adenotonsillectomy (A&T) remains the primary treatment for pediatric patients with OSAS. However, approximately 20–40% of cases do not achieve complete resolution even after the procedure [24,25,26]. An extensive longitudinal study by Huang et al. followed 135 children for 36 months after A&T, revealing that a significant proportion did not fully recover from the disease [27]. Astonishingly, 68% of the children experienced residual OSAS after undergoing A&T. Several other studies have also demonstrated incomplete resolution of OSAS following this surgical intervention [28,29]. Some researchers have even hypothesized that the persistence of OSA after A&T could be partially attributed to smaller mandible sizes in pediatric patients [30].

In conclusion, the enigmatic interplay between adenoids, tonsils, and the respiratory system intrigues medical researchers. Their constant exposure to microbial antigens and allergens highlights their vital role in the immune response. The complexities of airway constriction and the challenges in treating OSAS further emphasize the need for comprehensive research to identify optimal treatment strategies for ATH and its associated health complications. Understanding these underlying mechanisms will undoubtedly pave the way for improved healthcare and better outcomes for affected individuals.

### Adenoidal Tonsillar Hypertrophy (ATH) and Its Influence on Obstructive Sleep Apnea (OSA)

A common belief regarding ATH is that it is primarily a respiratory issue and that ATH, mouth breathing, and SDB there is lack of correlation between these conditions. In addition, ATH is perceived as a surgical issue that exclusively involves the respiratory system without considering the potential implications on facial morphology and craniofacial development.

**Figure 1:**
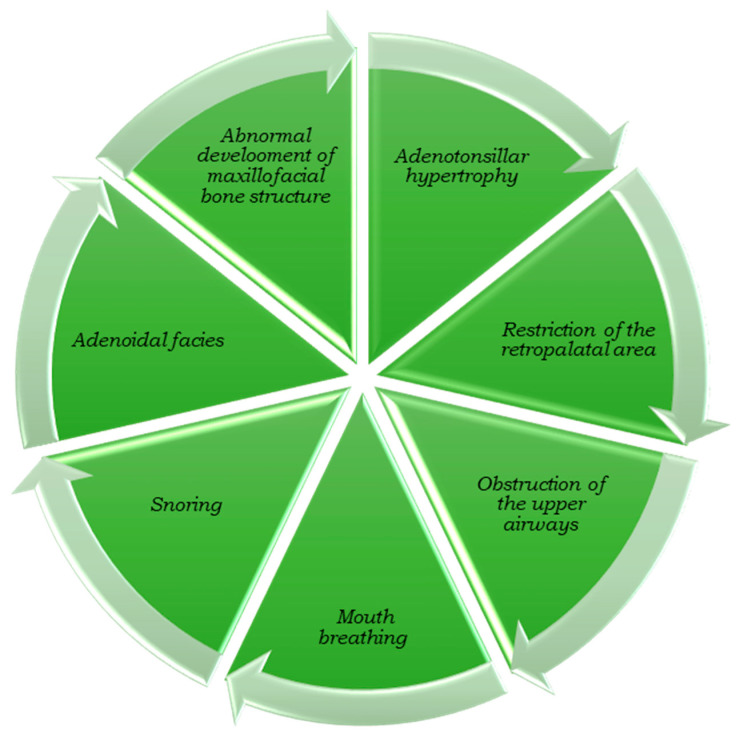
The progression from adenotonsillar hypertrophy to facial dysmorphism.

The impact of ATH on the development of OSA has been widely acknowledged in the scientific literature [31,32]. The available evidence concerning the origin of ATH has enhanced the comprehension of the fundamental mechanisms that trigger ATH’s onset. Notably, mouth breathing might have a substantial role in this progression. Inflammatory or infectious processes can trigger the growth of tonsillar or adenoid tissue, leading to upper airway obstruction, mouth breathing, and subsequent OSA (Figure 1).

Some beliefs may consider mouth breathing as a transient effect of ATH, without recognizing its potentially significant role in developing craniofacial features associated with SDB. However, formal evidence demonstrates that mouth breathing has relevant implications. Research has highlighted its important role in modifying facial morphology and the onset of craniofacial alterations, particularly about SDB.

Remarkably, individuals with tonsillar hypertrophy often exhibit distinctive facial characteristics, including increased mandibular ramus length, a more horizontally oriented growth pattern, increased mandibular body length, a more anterior mandibular position, and a minor sagittal discrepancy between the maxilla and mandible compared to individuals with adenoid hypertrophy [33]. Furthermore, chronic SDB problems can influence the facial structure and upper airways, leading to the phenomenon commonly known as “adenoid facies” [34]. The progressive enlargement of the adenoids specifically narrows the retropalatal area, a frequent site of obstruction within the upper airways [35,36].

The Enigma of Adenoid Facies:

Adenoid facies is characterized by the open-mouthed appearance seen in children, often accompanied by a slender nose, shortened upper lip, constricted palate, elevated palatal vault, and dental overcrowding [37]. Scientific studies have provided evidence supporting the connection between ATH and specific facial characteristics. Mouth breathing, snoring, and adenoid facies are frequently associated with ATH [38].

A common belief regarding snoring in children is that it is considered normal and transient. Often, snoring in children may be believed to be a temporary and harmless phenomenon without recognizing its potential link to SDB and possible associated craniofacial alterations. A study has demonstrated a correlation between the degree of nasal obstruction in children with primary snoring and changes in cephalometric parameters related to vertical craniofacial growth [39]. As ATH compels children to rely on mouth breathing as their primary mode of respiration, some researchers propose that prolonged mouth breathing can lead to abnormal development of the maxillofacial bone structure [14] (Figure 1). Furthermore, the very act of breathing plays a crucial role in the mechanism of pharyngeal collapse, an essential aspect of OSA [40].

In the study conducted by Villa et al. [41], a comparison was made between the adenoid phenotype and two sleep conditions: primary snoring and OSA. While seven patients (9.2%) with the adenoid phenotype exhibited primary snoring, 38 patients (18.7%) had OSA. However, the statistical examination did not uncover a noteworthy distinction between the two groups (*p* = not significant). This outcome may be attributed to the relatively small number of patients with the adenoid phenotype, leading to insufficient statistical power. Additionally, Bokov et al. [42] found that children with OSA and a mouth-breathing habit had a higher prevalence of adenoid facies than those with OSA but no mouth breathing.

Furthermore, children with a mouth-breathing habit showed a more severe form of OSA associated with increased pharyngeal compliance. On the contrary, children with OSA without mouth breathing did not demonstrate a significant increase in adenoid phenotype prevalence compared to children with only primary snoring (Villa et al.). Therefore, while there might be some disparity, the studies conducted by Villa et al. [41] and Bokov et al. [42] provide valuable insights into the association between OSA and adenoid facies, as well as the impact of mouth breathing on the severity of the condition.

Understanding the Link between ATH and OSA:

ATH is often identified as the primary cause of SDB. However, it is essential to recognize that the explanation for apneas cannot be solely attributed to adenoidal or tonsillar hypertrophy. For instance, mouth breathing could theoretically prevent some apneas in adenoidal hypertrophy with nasal obstruction. Similarly, adenoidal hypertrophy may obstruct the distal part of the pharynx due to pharyngeal muscle fatigue. As highlighted by Arens and Marcus [23], considering the contribution of abnormal neuromotor tone seems indispensable in understanding obstructive apneas. Therefore, it is crucial to emphasize better the role of abnormal neuromotor tone and adenoidal or tonsillar hypertrophy in explaining apneas.

The prolonged reliance on mouth breathing can affect the bone structure of the face and upper airways, potentially leading to abnormal development. Additionally, mouth breathing profoundly influences the mechanism of pharyngeal collapse, a pivotal element of SDB. Nevertheless, it is crucial to recognize that the current conclusions are drawn from a limited number of studies, and further research is indispensable to unravel the intricacies of the relationship between ATH and the development of abnormalities in the bone structure of the face and upper airways. Pursuing this knowledge will undoubtedly pave the way for enhanced understanding and improved management of ATH and its associated health implications.

## 3. Craniofacial Alterations

### Craniofacial Variations and Adenoidal Tonsillar Hypertrophy: Unraveling the Complex Relationship

**Figure 2:**
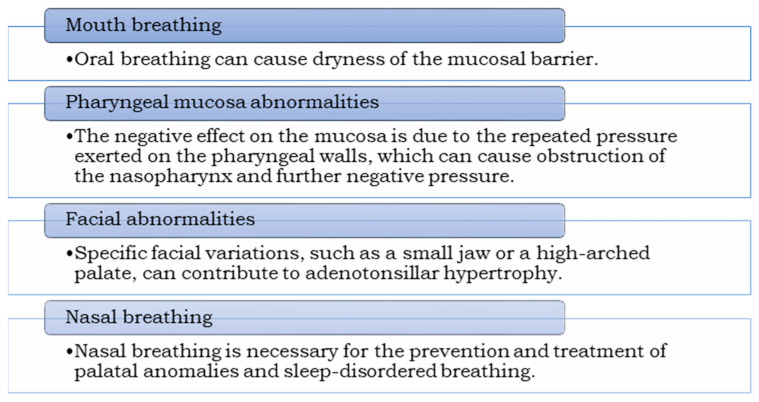
Effects of mouth breathing on respiratory health and correlation with ATH. Mouth breathing can lead to various negative consequences on respiratory health, such as tonsillar and adenoidal hypertrophy, and how nasal breathing can help prevent and treat these issues.

A common belief is that craniofacial features are not influenced by respiratory problems such as ATH and mouth breathing. This belief may underestimate the role of such conditions in shaping facial morphology and overlook the potential connection between SDB and craniofacial alterations. A fascinating alternative chain of events is proposed about ATH (Figure 2). This hypothesis suggests that mild facial alterations may play a pivotal role in the development of ATH. Kim and Guilleminault showed that 93.3% of children with SDB presented specific facial characteristics considered risk factors for OSA. These features include a small mandible and/or a high and narrow hard palate associated with a narrow nasomaxillary complex. These specific facial characteristics were present in 93.3% of children with OSA. However, the prevalence of OSA is not provided in this study [43].

Controversies Surrounding Mouth Breathing and SDB:

Mouth breathing has been linked to distinct craniofacial structures and OSA, yet its role in SDB remains controversial. On the one hand, breathing through the mouth can diminish the humidity of the mucosal barrier, increasing the susceptibility to infections, inflammation, and subsequent growth of tonsillar and adenoidal tissue [44,45]. This negative impact on the mucosa is attributed to the repetitive pressure exerted on its walls, leading to additional negative pressure and nasopharyngeal obstruction (Figure 2). Conversely, some studies have reported that children with mouth breathing exhibit more favorable development of the bony nasopharynx, mandibular length, and mandibular growth direction compared to those with OSA. Surprisingly, specific authors have concluded that despite mouth breathing, the anatomical morphology characterized by well-developed dentoalveolar structures and mild ATH may offer protection against OSA [46].

Pharyngeal Hypotonia and Craniofacial Abnormalities:

Research conducted by Yu-Shu Huang and Christian Guilleminault on pre-term-born children revealed that pharyngeal hypotonia, despite a typical hard palate at birth, can lead to SDB and alterations in the palate itself. Tonsillar enlargement was only observed in children with mouth breathing and a high, narrow hard palate, suggesting that the latter may result from mouth breathing [44]. Additionally, some authors propose that craniofacial abnormalities contribute to nasal obstruction, implying that facial variations could be the initial factors leading to the development of ATH and nasal obstruction [45,47].

Unraveling the Intricacies of the Connection:

In summary, common facial structural variations, such as an ogival palate, nasal or septal deformity, and a small jaw, might serve as the underlying causes of the onset of ATH rather than being its consequence [45]. The intricate relationship between craniofacial variations and SDB remains incompletely understood and can be influenced by multiple factors, including mouth breathing and pharyngeal hypotonia. Moreover, specific facial characteristics have been posited as potential causes rather than consequences of ATH. The meta-analysis did not find sufficient evidence to support a direct causal relationship between craniofacial structure and pediatric SDB. On the other hand, substantial evidence indicates reduced upper airway width in children with OSA. In particular, the reduced upper airway width in children with obstructive sleep apnea is a notable finding [48]. Further research is necessary to comprehensively understand the connection between craniofacial variations and SDB and develop appropriate preventive and therapeutic interventions. Pursuing this knowledge will undoubtedly pave the way for improved healthcare and management of ATH and its associated health complexities.

## 4. Intricate Factors Shaping Craniofacial Structure and Sleep-Disordered Breathing (SDB)

The development of skeletal and facial alterations is undoubtedly influenced by an amalgamation of diverse factors, ranging from genetics and diet to oral behavior and human development [49]. These intrinsic and extrinsic factors can come into play during fetal development and persist throughout an individual’s life, ultimately affecting the likelihood of developing OSA and related SDB conditions.

Exploring Theories on Craniofacial Structure and SDB:

In the quest to understand the relationship between craniofacial structure and SDB, two prominent theories have emerged the “gracilization theory” [50] and the “gravitational hypothesis” [51]. These theories offer distinct physiological mechanisms that underpin the association between craniofacial structure and SDB.

The Gracilization Theory:

The gracilization theory revolves around evolutionary diet and facial anatomy changes over time. It suggests that alterations in diet and lifestyle have contributed to the gradual refinement and “gracilization” of the facial structure. Such changes may influence the airway dimensions and respiratory mechanics, potentially leading to an increased susceptibility to SDB. As our ancestors adapted their diet and behavior, these shifts might have inadvertently influenced the development of craniofacial features contributing to SDB [52].

The Gravitational Hypothesis:

On the other hand, the gravitational hypothesis highlights the significance of body position during sleep and the role of gravity in affecting the upper airways. When individuals assume specific sleeping postures, the influence of gravity on the upper airway can potentially lead to airway collapse and obstruction, contributing to SDB. This theory postulates that the body’s positioning during sleep interacts with the anatomical features of the upper airway, shaping the development of SDB [52].

Comprehensive Understanding through Further Research:

As we delve deeper into the complexities of craniofacial structure and its connection to SDB, it is crucial to acknowledge that both theories offer valuable insights, and the relationship between craniofacial variations and sleep-related breathing conditions remains multifaceted. The interplay of genetic predisposition, dietary patterns, lifestyle choices, and body position during sleep undoubtedly contributes to the intricate web of factors that influence craniofacial development and the occurrence of SDB. Therefore, continuous research efforts are imperative to understand these mechanisms comprehensively and formulate practical preventive and therapeutic approaches for individuals affected by SDB conditions. By unraveling the mysteries surrounding craniofacial structure and SDB, we can strive towards improved healthcare and enhanced well-being for those grappling with these intricate health complexities.

### 4.1. Gracilization Hypothesis

The gracilization theory, put forth by Weber in 2023 [53], posits that changes in the human diet over time, as supported by Luca et al. in 2010 [54], have weakened the masticatory muscles. Consequently, this has influenced the craniofacial structure, reducing the volume of the upper airways. According to this theory, the mandible and tongue have evolved to be less voluminous to adapt to dietary shifts, including consuming solid foods and developing language. However, this reduction in tongue space within the oral cavity may contribute to airway obstruction during sleep, as discussed by Katz et al. in 2017 [55].

Additionally, the gracilization hypothesis proposes that the decrease in the robustness of the human skeleton and facial bones can be attributed to the transition from an active foraging lifestyle and a diet of raw foods to a more sedentary lifestyle and an agricultural diet. Lacruz et al. [56] support this notion, stating that this transition has reduced bone mass and decreased mechanical load on the jaws during chewing.

**Figure 3:**
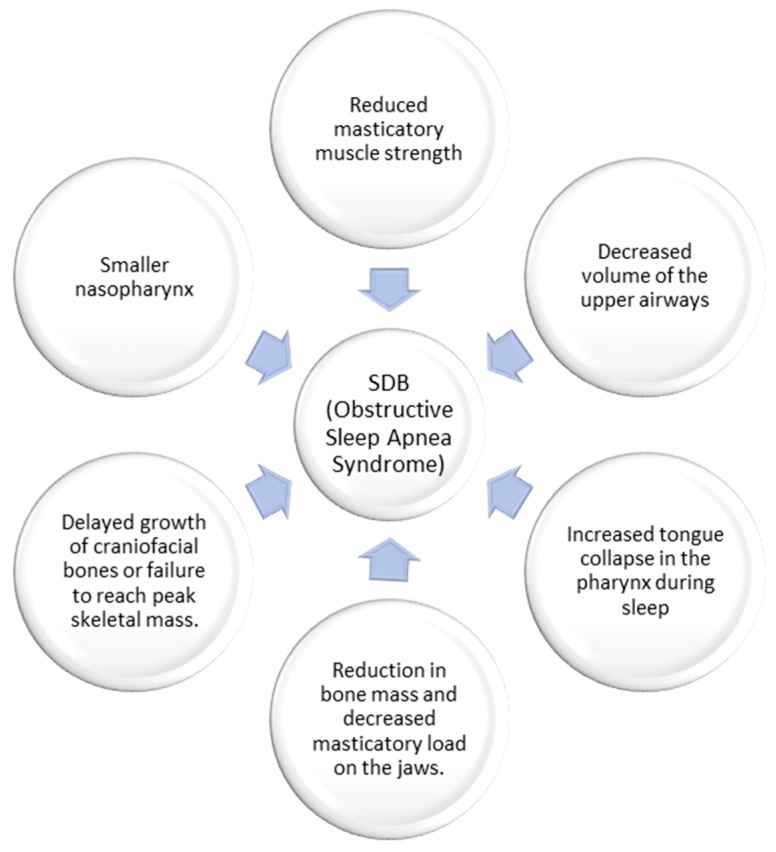
The hypothesis of gracilization on the genesis of sleep-disordered breathing.

Recognizing that these theories offer possible explanations for the connection between craniofacial changes and SDB is essential (Figure 3). However, further research is required to fully comprehend the intricate interplay between diet, craniofacial conformation, and the development of airway obstruction during sleep [49,57].

Eimar et al. conducted two cross-sectional studies to explore the impact of OSA on bone density, using Mandibular Cortical Width (MCW) as a measure. The studies revealed that children with OSA had lower MCW than controls, suggesting a potential link between OSA and bone density. The authors proposed that an inability to attain maximum skeletal mass or delayed growth of craniofacial bones might lead to a reduced nasopharynx size, potentially playing a role in the emergence of SDB [58]. Similarly, a study by Fernandes Fagundes et al. [59] observed reductions in MCW among children with OSA or those at high risk of OSA, indicating potential interactions between mandibular bone development and pediatric OSA.

Skeletal alterations and oral breathing can result in changes in the position of the maxillary palate and mandibular body relative to the mandibular ramus, leading to an increased gonial angle [60]. These changes can affect various structures, including the maxillary alveolar crest, the vomer, and the ethmoid, leading to septal deformities and retroclination of the pre-maxilla and its incisors. The development of OSA has been associated with a reduction in the size of the hard palate, which can subsequently decrease the available space for the tongue [43,61].

During sleep, the reduced retaining mechanism within the oral cavity can cause the tongue to collapse into the pharynx, increasing the likelihood of OSA worsening. This condition can further impede airflow and exacerbate symptoms of SDB [62].

In conclusion, the gracilization theory posits that changes in the human diet have reduced masticatory muscle strength, shaped the craniofacial region, and result in a decreased upper airway volume. This reduction in airway space increases the risk of tongue collapse during sleep, contributing to OSA. Additionally, some researchers propose that gracilization is linked to transitioning from a raw food-based diet to a sedentary lifestyle and agricultural diet, leading to reduced bone mass and jawbone load.

Furthermore, studies suggest that children with OSA exhibit lower bone density than those without the condition. This lower bone density could be attributed to the failure to achieve optimal skeletal mass or delayed craniofacial bone growth, leading to a smaller nasopharynx and consequent SDB.

These findings provide valuable insights into the intricate relationship between craniofacial anatomy, the human diet, and the development of OSA. However, further research is essential to comprehensively understand these factors’ complex interactions and develop practical preventive and therapeutic approaches for OSA.

### 4.2. The Impact of Gravity on Sleep-Disordered Breathing: Unraveling the Complexity

Sleep, a fundamental aspect of human existence, is a physiological state during which the body undergoes various intricate processes. Among them, the loss of muscle tone during sleep can lead to the collapse of the upper airways, giving rise to conditions such as apnea and snoring [63]. However, the influence of gravity on sleep-related breathing disorders (SDB) is a captivating facet that warrants exploration.

**Figure 4:**
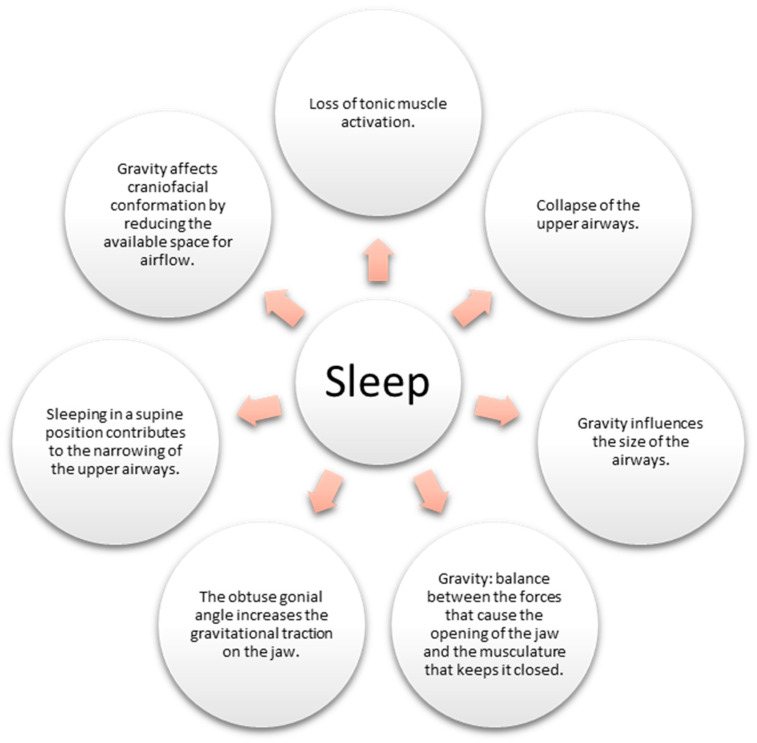
Impact of gravity on the airways during sleep.

Gravity exerts its force on the body, mainly when one adopts a supine position (lying on the back) during sleep. This positioning can trigger increased compression and narrowing of the upper airways, accentuating the likelihood of SDB [64]. Remarkably, the impact of gravity extends beyond mere airway mechanics, extending its grasp to influence craniofacial conformation, consequently contributing to SDB (Figure 4).

Gravity also orchestrates the ballet of oral breathing, regulating the interplay of forces determining jaw movement. The delicate balance between the forces promoting jaw opening and those ensuring its closure is influenced by this gravitational pull [65]. Studies have suggested that an obtuse gonial angle could favor oral breathing by increasing the gravitational force on the jaw [65].

Intriguingly, investigations conducted on astronauts in gravity-deficient environments have unveiled a positive influence on sleep-related breathing. The absence of gravity in such settings correlates with reduced apnea and snoring incidents [66].

To comprehend the pathophysiology of SDB disorders comprehensively, it is imperative to acknowledge the combined effect of muscle tone loss and gravitational influence on the collapse and constriction of the upper airways [63]. Moreover, gravity’s impact on the human body is far from uniform, varying considerably based on individual body position and anatomical structure. Thus, a comprehensive exploration of gravity’s influence on the airways and sleep necessitates further research and investigation [64].

In conclusion, the intricate relationship between gravity and SDBs is a captivating study area that illuminates human physiology’s complexities during slumber. Understanding the interplay between muscle tone loss, gravity’s influence on the upper airways, and its impact on oral breathing is crucial in comprehending the pathophysiology of SDB. This research offers a compelling glimpse into the role of gravity in shaping the dynamics of the respiratory system during sleep, paving the way for future investigations to unravel the mysteries that still lie in the realm of slumber.

## 5. Conclusions

The present study aimed to investigate the implications of ATH on facial morphology and its impact on facial shape. The adenoid facies, characterized by a narrow nose, shortened upper lip, narrow palate, high palatal arch, and dental crowding, is commonly associated with ATH. Extensive research has indicated a correlation between the degree of nasal obstruction in children with primary snoring and alterations in cephalometric parameters associated with vertical craniofacial growth. The pathogenesis of ATH has garnered considerable interest, with some researchers hypothesizing that prolonged mouth breathing, a common consequence of ATH, may contribute to the anomalous development of the facial bone structure.

Additionally, this study aimed to explore the correlation between craniofacial conformation and the severity of SDB in individuals with ATH. Children with SDB presented specific facial characteristics considered risk factors for OSA. These features included a small mandible and/or a high and narrow hard palate associated with a narrow nasomaxillary complex. Furthermore, mouth breathing has been associated with specific craniofacial structures and OSA, although its role in SDB remains a topic of debate. Some studies suggest that mouth breathing may negatively impact the nasal mucosa, contributing to nasopharyngeal obstruction and subsequent SDB. On the other hand, other authors hypothesize that craniofacial abnormalities may contribute to nasal obstruction, implying that facial variations could be the initial factors leading to the development of ATH and nasal obstruction.

This study also delved into two theories to elucidate the complex relationship between craniofacial conformation and SDB in ATH. The “gracilization theory” focuses on the evolution of diet and changes in facial anatomy over time, suggesting that dietary and behavioral adaptations throughout human history may have influenced the development of craniofacial features associated with SDB. Conversely, the “gravitational hypothesis” highlights the importance of body position during sleep as a factor interacting with upper airway anatomy, thereby contributing to the onset of SDB. Both theories offer intriguing perspectives on the intricate relationship between craniofacial conformation and SDB in individuals with ATH.

Addressing this complex relationship between ATH, mouth breathing, and craniofacial changes requires further investigation. Gaining a more comprehensive insight into the pathophysiological mechanisms that underscore these associations is crucial. Unraveling the risk factors associated with the failure of A&T is crucial, as it can shed light on the long-term effects of ATH on craniofacial growth. Additionally, developing new diagnostic techniques that enable early detection of upper airway compromise is imperative to facilitate timely interventions.

Innovative therapeutic approaches should also be explored for patients who do not respond favorably to surgical treatment. These may include positional therapy, which aims to alter sleep positions to improve breathing, and orofacial myofunctional therapy, designed to address and correct improper oral and facial muscle functions.

To sum up, this research offers valuable perspectives on the diverse dimensions of how ATH influences facial morphology and its correlation with SDB. Comprehending the relationship between craniofacial characteristics and the severity of SDB could hold clinical significance for timely diagnosis and treatment strategizing in pediatric patients with ATH. Further research in this area will contribute to a comprehensive comprehension of the underlying mechanisms and aid in developing targeted therapeutic interventions for individuals affected by these conditions.

## Data Availability

Not applicable.
